# Visual Acceleration Perception for Simple and Complex Motion Patterns

**DOI:** 10.1371/journal.pone.0149413

**Published:** 2016-02-22

**Authors:** Alexandra S. Mueller, Brian Timney

**Affiliations:** Department of Psychology, University of Western Ontario, London, Ontario, Canada; Monash University, AUSTRALIA

## Abstract

Humans are able to judge whether a target is accelerating in many viewing contexts, but it is an open question how the motion pattern *per se* affects visual acceleration perception. We measured acceleration and deceleration detection using patterns of random dots with horizontal (simpler) or radial motion (more visually complex). The results suggest that we detect acceleration better when viewing radial optic flow than horizontal translation. However, the direction within each type of pattern has no effect on performance and observers detect acceleration and deceleration similarly within each condition. We conclude that sensitivity to the presence of acceleration is generally higher for more complex patterns, regardless of the direction within each type of pattern or the sign of acceleration.

## Introduction

Outside the laboratory, objects and observers rarely move at constant speeds, so it is important to be able to process changes in visual motion in order to intercept or avoid objects and to accomplish voluntary tasks [1]. Although humans are relatively insensitive to changing speed [2–4], we are still able to perceive visual acceleration under a wide variety of viewing conditions. For example, when looking straight ahead, forward motion creates radial expansion in the visual image, whereas backward motion produces radial contraction. We also tend to see translation or lateral flow when moving sideways (lateral head motion) or when visually pursuing an object moving across our visual field. In addition, we may experience rotational motion as our heads tilt. Each of these forms of motion differs in terms of visual complexity; for instance, in radial optic flow visual elements move simultaneously in all cardinal and oblique directions, whereas in translation all of the elements move in the same direction. However, there is only limited information available on whether the ability to detect acceleration is influenced by the global motion pattern, because most experiments on acceleration perception have used stimuli moving in the fronto-parallel plane (i.e., translation). Those studies that have investigated the influence of pattern type on motion perception in general (i.e., not specific to acceleration perception) have produced mixed results [5–14].

It has been proposed that the visual system reduces simple and complex motion patterns within the visual image into their underlying primary forms (i.e., translational, rotational, and radial patterns), which can be further decomposed into local spatiotemporal components [15,16]. If the visual system were to solely rely on this method of motion analysis, one might expect that we should process visual motion similarly across patterns that vary in complexity. However, using a motion coherence task, Lee and Lu [14] tested stimuli composed of small Gabor elements (moving at the same speed across the display) that were arranged in such a way as to present a global pattern of translational, rotational, or radial motion. They found that observers perform better when viewing complex motion patterns (rotational and radial) than simpler motion patterns (translational), which suggests that the visual system does not uniformly apply the same process of motion analysis to distinct motion patterns.

There is considerable support in the literature for a motion processing hierarchy in the primate visual system, as simpler aspects of motion tend to be processed relatively earlier in the visual pathway than more complex features [17–20]. Although earlier areas, such as V1, process fundamental spatiotemporal components of the visual image [21–23], the existence of a functional hierarchy suggests that those local signals are pooled by specialized higher-order areas devoted to processing more complex aspects of motion. For example, the coding of complex motion patterns occurs in areas such as MST by neurons that have larger receptive fields tuned to specific patterns, such as radial, translational, and rotational motion [24–28]. Moreover, radial optic flow contains information about heading, which is processed by areas such as MST and VIP [29–32]. Based on those findings and despite our general insensitivity to visual acceleration, perhaps those higher-order areas may be recruited to process radial acceleration, but not horizontal acceleration, because of the self-motion cues in the optic flow pattern. This recruitment would be consistent with the finding that MST shows greater activation in response to radial optic flow than to translation [33]. Although one might therefore expect better acceleration detection for radial optic flow than for horizontal translation, it is unclear whether such an asymmetry actually exists.

The aim of this study was to investigate whether our ability to detect the presence of acceleration is different for radially and horizontally moving random dot patterns. However, radially moving dot stimuli that are designed to resemble our experience with motion in a three-dimensional environment contain dot density, size, and speed gradients (i.e., where the density, size, and speed of the dots vary as a function of eccentricity from the focus of optic flow). For the purpose of our study this presents a problem, because translating random dot arrays do not contain dot density, size, or speed gradients and it is possible that motion perception may be affected by some or all of these factors [11,34–39]. Therefore, in the current paper we held these parameters constant across the display in order to determine whether the visual complexity of a motion pattern *per se* influences the ability to detect the presence of acceleration. This level of control came at a cost of the realism of our radial optic flow displays, but by holding these dot parameters constant observers could not distinguish which stimulus belonged to which motion pattern condition based on a single frame. In addition, many earlier studies on the effect of pattern type on motion perception have had similar deviations in ecological validity in their stimulus designs in order to make direct comparisons between conditions [5,7,8,13,14]. Likewise, as it is not possible to keep both the motion profile and synchrony constant across the display when presenting rotational motion, because dots travel shorter distances in the centre than in the periphery, we did not test rotational acceleration in the current study.

Another unresolved issue is whether acceleration detection differs between radial expansion and contraction. It could be argued that due to our familiarity with expansion over contraction, because we move forward more often than backward, we may be more sensitive to the presence of acceleration in expanding than contracting optic flow. However, the psychophysical evidence is mixed as to whether a radial anisotropy exists in our general sensitivity to motion [8–11,13,40,41]. Consequently, in the present study we also manipulated direction within the radial and horizontal motion conditions.

## Materials and Methods

### Participants

Seven individuals (including author ASM) with an average age of 26.3 years (*SD* = 2.43 years) comprised the sample, and five were female. As this was a difficult task, participants were pretested to ensure that they could perform reliably and were willing to commit to the extensive testing required. All observers had normal or corrected–to–normal visual and stereo acuity. Individuals provided written informed consent and were reimbursed $20 for their participation. This experiment was conducted in accordance with institutional regulations and the Declaration of Helsinki and was approved by the University of Western Ontario’s Non-Medical Science Research Ethics Board (NMREB file number: 104932).

### Stimuli and Apparatus

Graphics and psychophysics software (VPixx, version 3.14, VPixx Technologies Inc., Saint-Bruno, Quebec, Canada [42]) was used to generate and present the stimuli on a 51.5 cm LaCie electron22blue II monitor (Mitsubishi Electric Corporation, Japan) with a 120−Hz refresh rate and display resolution of 1024 x 768 pixels. The random dot stimuli were white square-shaped dots subtending 0.1° x 0.1° on a black background. Dot position was updated every frame and dot size remained constant throughout the visual field. The arrays moved continuously and 100% coherently from behind an invisible (i.e., the border was not defined) stationary aperture that was 37° x 27° (width x height) at a viewing distance of 60 cm centered in the middle of the screen.

In the horizontal motion patterns, dots moved either leftward or rightward. The dots in the radial motion patterns moved either away from (expansion) or towards (contraction) the center of the display. Leftward, rightward, expanding, and contracting motion represented four separate conditions. Although we did not expect differences in performance between the leftward and rightward motion conditions, we included the manipulation to balance the experimental design. Within every subsequent frame the dots were displaced by the same amount across the visual field, which corresponded to the stimulus’ speed divided by the frame rate. In the horizontal motion conditions, the dots moved in the same direction for a given stimulus. In contrast, for the radial motion conditions the direction of displacement was contingent on the dot’s location in the stimulus. Specifically, dots were displaced along the vector from the centre or the periphery of the stimulus to the current position in that given frame, which resulted in dots streaming inward (contraction) or outward (expansion) from the centre of the display. The speed of every dot increased or decreased over the course of the presentation according to the rate of acceleration or deceleration, respectively, for that particular trial.

Average dot density was constant at 0.75 dots/deg^2^ in every frame for all conditions. In the first frame at the beginning of every stimulus’ presentation, a set of dots was generated and placed with an average uniform distribution of random locations within the aperture. For the horizontal motion conditions, dots were replaced in random vertical locations along the border of the aperture opposite to the direction of motion when they disappeared outside the visible area of the aperture. For the radial expansion conditions, the VPixx program partitioned the display into eight uniformly spaced eccentricities and eight uniformly spaced meridians at 45° intervals. As a result, there were 64 truncated annuli centered on the focus of expansion (i.e., in the middle of the display). In every frame, the software calculated the instantaneous density of dots within each annulus and then calculated a low-pass filtered time-averaged density that was equal to half of the instantaneous density of the frame plus half of the previous frame’s time-averaged density. When a dot reached the border of the aperture, it was replaced by a new dot in a random location within the truncated annulus that contained the lowest time-averaged density, thus keeping the average dot density constant between frames. For the radial contraction conditions, the software generated radially expanding patterns that were played backwards to the observer, while preserving the sign of acceleration between both types of patterns, in order to keep the dot density constant and the motion pattern equivalent between the two radial direction conditions. Several earlier studies have used similar methods for designing radial motion stimuli [33,43–45].

In addition, we also manipulated the sign of acceleration while adopting an approach used in several previous studies [46–48] that controls for the possibility that acceleration and deceleration detection might differ because the speed ranges for each condition are not the same. Specifically, we held average speed constant between the acceleration and deceleration conditions, rather than using the same initial or final speeds. To do this, the motion profile of each dot in all four motion direction conditions was centered on 10 deg/s, which was the midpoint speed between the starting and final speeds of every stimulus, using the following equation:

speed=10+a(t−(ttotal2)), (1)

where variable *a* represents the acceleration/deceleration rate, *t* refers to time, and *t*_*total*_ is the total presentation duration (750 ms). Stimuli that accelerated or decelerated did so continuously for the entire presentation.

### Procedure

We used a two-interval forced choice (2IFC) task with the method of constant stimuli. For each condition there were 7 comparison rates of acceleration and deceleration, drawn from a possible range of ±1 to 10 deg/s^2^, in steps of ±1 deg/s^2^. The actual range selected for an individual participant depended on how well he or she was able to do the task. The standard stimulus moved at a constant speed of 10 deg/s. In every trial a comparison stimulus and a standard stimulus were presented one after the other in random order.

Participants were tested in the dark and viewed the screen binocularly, using a chin rest to minimize head movements. At the beginning of every trial a red fixation target with the shape of a 0.5° diameter crosshair against a black background was presented for 500 ms. Participants were told to fixate the crosshair target at the beginning of every trial until it disappeared, then they were free to track the random dot stimuli. Once the fixation target disappeared, the standard or comparison stimulus was immediately presented for 750 ms, followed by a black screen for 500 ms, and then the standard or comparison stimulus for another 750 ms. The task was to identify which stimulus was changing speed, and participants were informed that one stimulus would change speed either positively (accelerate) or negatively (decelerate) in every trial. They indicated their decision by pressing a key on a keyboard. Trials were self-paced, initiated by pressing the spacebar. An audible beep followed each key and spacebar press.

Accelerating and decelerating comparison stimuli were randomly interleaved across trials within the four motion direction conditions, and participants completed one condition at a time. The order of the conditions and acceleration/deceleration rates within each condition was randomized. The task required discriminating changing speed from constant speed while monitoring the sign of acceleration, and the cognitive load would explain why observers reported that the task was challenging. In an unpublished earlier version of this study, we blocked acceleration and deceleration separately and participants reported that that task was much easier than the one used in the current study with randomly interlaced acceleration and deceleration conditions; however, performance and the effect of pattern type were very similar for both types of tasks. In the current study, most participants said that they tended to expect either acceleration or deceleration, but not both, despite task instructions that they would be randomly interlaced throughout the experiment. Nonetheless, with enough practice they began to perform reliably. Participants were given at least 80 practice trials prior to the experimental task. They completed at least two experimental runs per condition, each containing 10 trials per acceleration/deceleration rate, for a total of 140 acceleration and 140 deceleration trials for each motion direction condition. Due to performance variability in the initial trial runs, we included only the last two runs for analysis that, when combined, produced non-significant Pearson goodness of fit coefficients (as determined through Chi-square analyses) for the probit regression used to interpolate threshold rates.

### Analysis

The data were analyzed using SPSS software (IBM Corporation, Armonk, NY). Table 1 shows the mean absolute value 75% correct detection threshold rate for each condition.

**Table 1:** Mean Absolute Value 75% Correct Acceleration and Deceleration Detection Threshold Rates (deg/s^2^).

	Acceleration Rate	Deceleration Rate
**Condition**	***M* (*SEM*)**	***M* (*SEM*)**
Left	5.12 (0.43)	5.35 (0.88)
Right	5.36 (0.50)	5.52 (0.92)
Expansion	3.55 (0.33)	3.72 (0.42)
Contraction	3.40 (0.63)	3.98 (0.62)

In order to make these results comparable to those of other studies in the literature, we transformed the data to show relative differences in performance for each condition. We used a similar procedure to that used by others [46–48] to make the thresholds functionally equivalent to Weber fractions (in percent), as shown in *Eq 2*:

relativethreshold=((vmax−vmin)((vmax+vmin)2))100, (2)

where *v*_*max*_ and *v*_*min*_ are the maximum and minimum speeds belonging to each detection threshold rate. The transformed data are expressed as the threshold speed difference (in percent) between the maximum and minimum speeds of an accelerating/decelerating stimulus relative to the speed of the standard stimulus needed to detect the presence of acceleration/deceleration, because the speed of the standard stimulus was the same as the average speed of the comparison stimuli within every condition. This linear transformation produces the same pattern of results observed in the absolute value detection threshold data.

## Results

**Fig 1:**
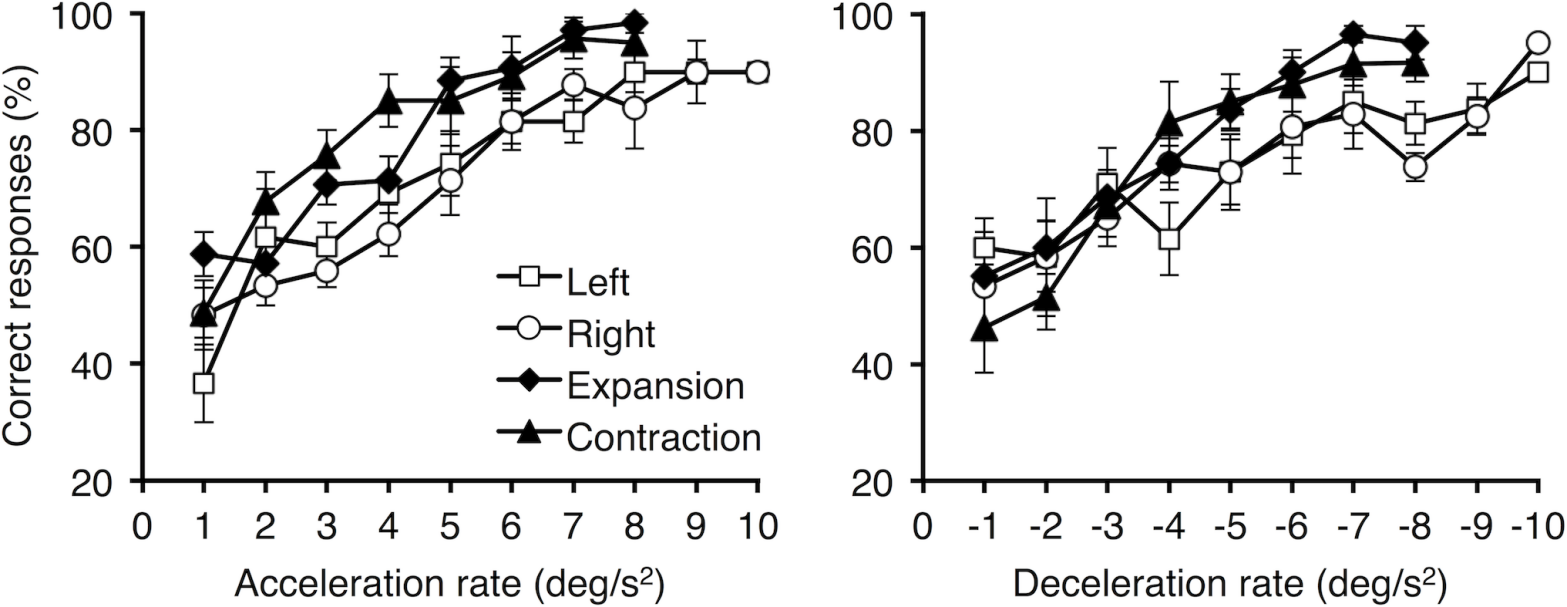
Mean percentage of correct response trials as a function of pattern direction. Acceleration conditions on left and deceleration conditions on right. Error bars are ± 1 *SEM*.

Fig 1 shows the performance in each condition, averaged across all participants. As expected, there were no apparent differences between the leftward and rightward motion conditions. Nevertheless, the curves for translation were shifted to the right, indicating that performance was better for radial motion, with no evident differences between the expansion and contraction conditions. In addition, the pattern of results was very similar for the acceleration and deceleration conditions.

To confirm these observations statistically, we first conducted a 2(pattern type) x 2(sign of acceleration) repeated measures ANOVA on the absolute value slopes of the psychometric functions acquired from the probit regression analysis used to obtain each participant’s 75% correct detection threshold rates. Then we performed a 2(direction) x 2(sign of acceleration) repeated measures ANOVA on those data for the radial and horizontal motion conditions separately. None of the data reported in this paper violated the sphericity assumption and therefore no corrections were necessary. Only the main effect of pattern type was statistically significant, *F*(1, 6) = 19.34, *p* = 0.01, η^2^_p_ = 0.76, as the slopes were steeper in the radial motion condition than in the horizontal motion condition (Table 2). There were no differences in the slopes obtained for the acceleration and deceleration conditions, *F*(1, 6) = 0.07, *p* = 0.81, and there was no effect of direction within the horizontal, *F*(1, 6) = 0.17, *p* = 0.70, or radial motion conditions, *F*(1, 6) = 0.52, *p* = 0.50. In addition, there were no interactions between the experimental variables.

**Table 2:** Mean Slopes (Absolute Values) of the Psychometric Functions.

	Acceleration	Deceleration
**Condition**	***M* (*SEM*)**	***M* (*SEM*)**
Left	0.23 (0.04)	0.23 (0.04)
Right	0.23 (0.02)	0.21 (0.06)
Expansion	0.33 (0.04)	0.30 (0.03)
Contraction	0.35 (0.06)	0.36 (0.09)

**Fig 2:**
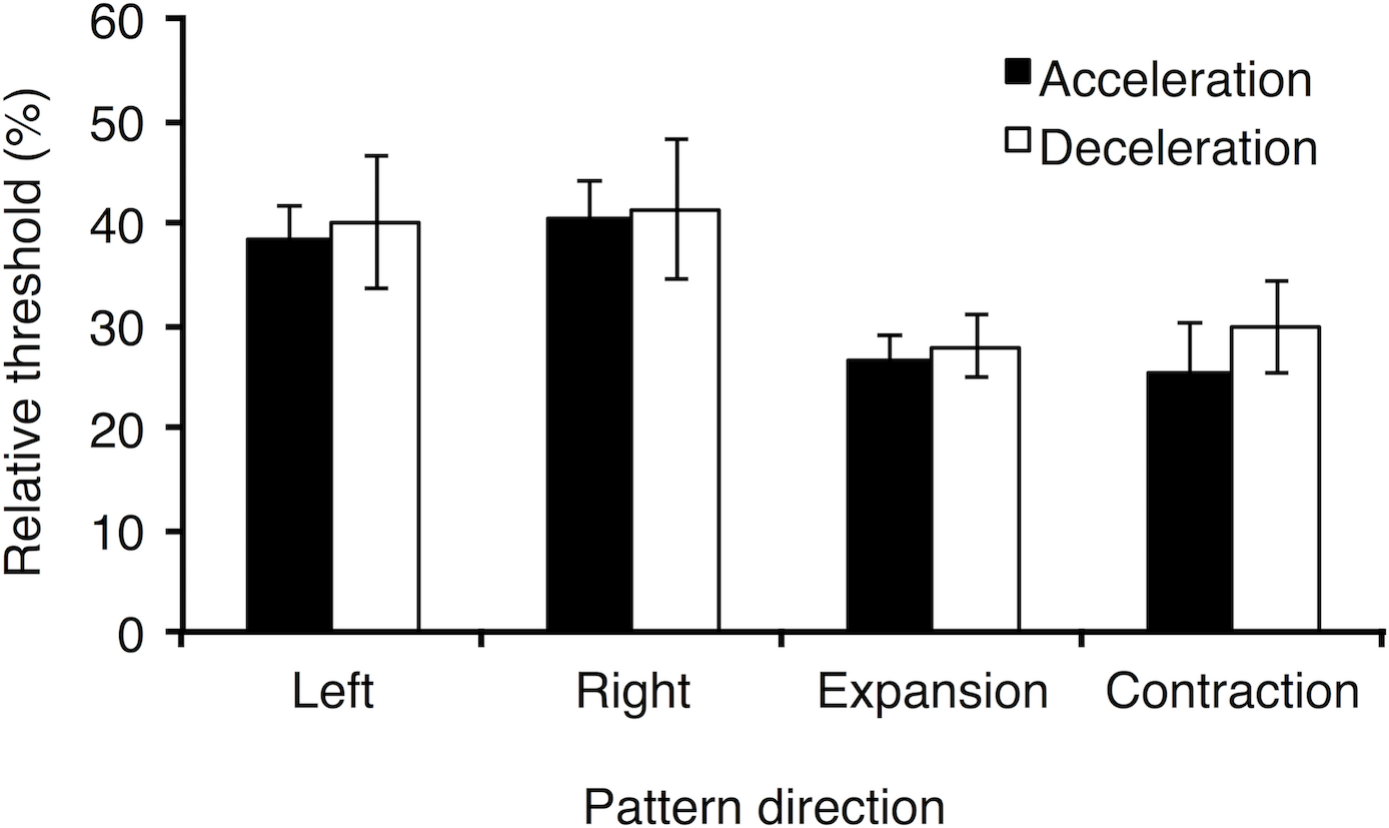
Mean relative acceleration and deceleration detection thresholds (%) as a function of pattern direction. Error bars are ± 1 *SEM*.

We then conducted the same repeated measures ANOVAs on the relative threshold data. The analysis produced a main effect of pattern type on detection accuracy, *F*(1, 6) = 22.98, *p* = 0.003, η^2^_p_ = 0.79, showing that detection thresholds were lower for radial motion than for horizontal motion (Fig 2). However, there were no statistically significant differences between the acceleration and deceleration conditions, *F*(1, 6) = 0.39, *p* = 0.56, for either type of motion pattern, *F*(1, 6) = 0.04, *p* = 0.86, nor were there any effects of direction for either the horizontal, *F*(1, 6) = 1.09, *p* = 0.34, or radial motion conditions, *F*(1, 6) = 0.03, *p* = 0.87. There were also no interactions between the experimental variables.

## Discussion

Our findings indicate that sensitivity to the presence of acceleration depends on the visual complexity of the motion pattern viewed. Specifically, observers are better at detecting acceleration in radial optic flow than in horizontal translation, although direction within each type of pattern has no effect. In addition, although observers detect the presence of acceleration and deceleration similarly within each motion direction condition, they show a general insensitivity to both as evidenced by high detection thresholds in every condition. These large thresholds are consistent with the acceleration detection performance reported in earlier studies [46–51]. Humans are far less sensitive to acceleration than to constant speed, as Weber fractions for speed discrimination have been reported to be between 4 and 7% over a wide range of stimulus parameters [52–57], whereas thresholds for acceleration detection or discrimination tend to be considerably higher. For example, Brouwer et al. [46] reported that at least a 25% difference between the initial and final speeds is needed to detect visual acceleration, although other studies have reported Weber fractions or relative thresholds that are much larger (e.g., [47]). This relative insensitivity to visual acceleration has been attributed to the fact that the primate visual system does not appear to contain cortical neurons that are sensitive to the rate of acceleration directly, whereas visual motion processing areas have neurons tuned to ranges of constant speed and direction [58–60].

According to Gibson [61], the more ecologically valid the visual stimulus is, the more representative the psychophysical performance should be in an experiment because humans are probably hardwired in some fashion to perceive motion better in certain types of stimuli than others. However, although radial motion is more visually complex than translation, we tend to see both types of motion patterns regularly in the natural environment. Moreover, our radial motion stimuli in this study did not contain depth cues with respect to dot size, density, and speed gradients and, as a result, were not consistent with the radial optic flow patterns that we tend to see outside the laboratory. Although Lee and Lu [14] used stimuli that were very different from ours, they too reported a complexity bias in motion coherence perception even though their radial motion stimuli likewise did not contain depth cues. They argued that using such stimuli would have predicted the opposite pattern in performance if the ecological validity of a stimulus alone were responsible for how we perceive motion. Nevertheless, perhaps the absence of depth cues in our radial motion displays may explain why we did not observe a difference between the expanding and contracting conditions, which should be taken into consideration for future studies on radial anisotropies in motion perception. Even so, Freeman and Harris [12] demonstrated a radial bias in motion detection performance when using radially moving random dot stimuli that contained speed gradients, which shows that our findings are not specific to radial stimuli without depth cues.

Furthermore, we note that most participants in this study reported experiencing vection in the radial motion conditions (i.e., the sensation of moving forward with the expanding stimuli and backward with the contracting stimuli). Palmisano, Allison, and Pekin [62] found that optic flow displays containing random acceleration of self-motion (i.e., jittering or oscillating motion profiles on the vertical and horizontal axes) elicit stronger impressions of vection than those without, which suggests that acceleration may be an important aspect of the ecological validity of optic flow, even for stimuli as controlled as ours. Therefore, our finding of a radial bias in acceleration detection might indicate that our stimuli were successful in eliciting the recruitment of higher-order areas in the visual system to increase the gain in processing acceleration in radial motion as compared to in horizontal translation. As the rate of radial optic flow should help to inform the observer about his or her rate of movement through the environment [63], having a predisposition to detect the presence of acceleration in radial motion may have implications for locomotion.
